# Coping Style as a Predictor of Relapse and Functioning in First Episode Psychosis

**DOI:** 10.1093/schbul/sbag079

**Published:** 2026-08-24

**Authors:** Anna Georgiades, Ryan Hammoud, Shangqing Liu, Maria Chiara Del Piccolo, Aljawharah Almuqrin, Alina Jorovat, Nandinee Rajkhowa, Stefania Tognin, Andrea Mechelli

**Affiliations:** Department of Psychosis Studies, Institute of Psychiatry, Psychology, and Neuroscience (IoPPN), King’s College London, London SE5 8AF, United Kingdom; CNWL, NHS Foundation Trust, Brent Early Intervention Service, 27-29 Fairlight Avenue, London NW10 8AL, United Kingdom; Department of Psychosis Studies, Institute of Psychiatry, Psychology, and Neuroscience (IoPPN), King’s College London, London SE5 8AF, United Kingdom; Department of Psychosis Studies, Institute of Psychiatry, Psychology, and Neuroscience (IoPPN), King’s College London, London SE5 8AF, United Kingdom; College of Artificial Intelligence Medicine, Chongqing Medical University, Chongqing 400016, China; Department of Psychosis Studies, Institute of Psychiatry, Psychology, and Neuroscience (IoPPN), King’s College London, London SE5 8AF, United Kingdom; Department of Psychosis Studies, Institute of Psychiatry, Psychology, and Neuroscience (IoPPN), King’s College London, London SE5 8AF, United Kingdom; Department of Health Sciences, College of Health and Rehabilitation Sciences, Princess Nourah Bint Abdul Rahman University, Riyadh, Saudi Arabia; Department of Psychosis Studies, Institute of Psychiatry, Psychology, and Neuroscience (IoPPN), King’s College London, London SE5 8AF, United Kingdom; CNWL, NHS Foundation Trust, Brent Early Intervention Service, 27-29 Fairlight Avenue, London NW10 8AL, United Kingdom; Department of Psychosis Studies, Institute of Psychiatry, Psychology, and Neuroscience (IoPPN), King’s College London, London SE5 8AF, United Kingdom; Department of Psychosis Studies, Institute of Psychiatry, Psychology, and Neuroscience (IoPPN), King’s College London, London SE5 8AF, United Kingdom; Department of Psychosis Studies, Institute of Psychiatry, Psychology, and Neuroscience (IoPPN), King’s College London, London SE5 8AF, United Kingdom

**Keywords:** coping style, first episode psychosis, positive symptoms, predictor, relapse

## Abstract

**Background:**

Compromised coping responses to life stressors may influence the development, maintenance, and recovery of psychosis.

**Study Design:**

A longitudinal design assessed 269 First Episode Psychosis (FEP) patients at baseline, 4, 8, and 12 months. Linear/logistic regressions and machine learning techniques evaluated whether baseline coping styles predicted clinical and functional outcomes.

**Study Results:**

Relapse occurred in 22 (9%), 35 (14%), and 50 (20%) participants within 4, 8, and 12 months, respectively. Maladaptive coping was associated with higher odds of relapse within 12 months (OR = 1.06, 95% CI: 1.00–1.12), hallucinations (OR = 1.07, 95% CI: 1.01–1.13) and delusions (OR = 1.07, 95% CI: 1.02–1.13), and with poorer Global Assessment of Functioning symptom and disability scores across follow-up. Adaptive coping was significantly associated with lower odds of hallucinations (OR = 0.96, 95% CI: 0.92–1.00) and delusions (OR = 0.95, 95% CI: 0.91–0.99) and with better functioning at 4 months (B = 0.35, SE = 0.13). Machine learning indicated that maladaptive (AUC = 0.770, accuracy [ACC] = 0.750) and adaptive coping (AUC = 0.729, ACC = 0.696) were significantly associated with relapse within 12 months, with maladaptive coping showing stronger predictive accuracy. Key contributors included self-distraction and substance use for maladaptive coping, and spiritual/religious coping and active problem-solving for adaptive coping.

**Conclusions:**

Baseline coping styles were associated with clinical and functional outcomes in FEP. These findings suggest that targeting maladaptive coping, particularly self-distraction and substance use, while promoting active problem-solving and spiritual/religious coping, may inform future intervention development aimed at improving symptom trajectories and long-term functional recovery.

## Introduction

Stress has repeatedly been implicated in the onset and exacerbation of psychosis symptoms.^1^^,^^2^ Individuals with psychosis have demonstrated heightened emotional reactivity to stress, which has been associated with greater positive symptom severity.^2–4^ Coping strategies in response to stress are therefore particularly relevant to this population (see Table 1 for coping-related definitions). There is growing evidence suggesting that responses to stress may play a critical role in the development, maintenance, and recovery of psychosis.^2^ Individuals at Clinical High Risk (CHR) for psychosis and those with established Schizophrenia (SZ) have been found to employ Maladaptive Coping Strategies (MCS) more often than Adaptive Coping Strategies (ACS).^5–7^ To date, only one longitudinal study has examined coping in CHR individuals over a 12-month period.^8^ This study found that MCS were associated with increased positive and negative symptoms, while ACS were associated with reduced symptom severity and greater social functioning.^8^ Evidence in First Episode Psychosis (FEP) is more limited. In this group, greater use of emotion-focused coping and lower use of problem-focused coping have been associated with higher perceived stress.^9^ Individuals with FEP have also demonstrated elevated levels of perceived stress along with a reduced tendency to employ ACS.^10^ Reduced adaptive coping has further been linked to higher allostatic load, a marker of cumulative biological stress burden, which in turn has been associated with more severe positive symptoms and poorer psychosocial functioning.^11^^,^^12^ Together, these findings suggest that coping strategies may be relevant to both psychological and biological stress processes in FEP.

**Table 1:** Definitions for Coping Terms.

**Coping Term**	**Definition**
Coping Strategy	The cognitive and behavioural efforts used to manage stress^1^
Emotion-Focused Coping	A strategy used to manage the emotional distress caused by a stressful situation, rather than addressing the situation itself. Strategies include seeking social support or social withdrawal, avoidance, practicing meditation, engaging in relaxation techniques, positive reframing (reframing the situation in a more positive light), and talking to others^1^
Problem-Focused Coping	Strategies aimed at directly addressing or resolving the source of stress, with the goal of reducing or eliminating it^1^
Maladaptive Coping Strategies	Behaviours or thought patterns that may provide temporary relief but lead to negative health outcomes, such as avoidance, rumination, and suppression^2^
Adaptive Coping Strategies	Behaviours or thought patterns that lead to positive health outcomes, such as reappraisal and distraction^2^
Reappraisal	An emotion regulation strategy that involves reframing the meaning of a situation to alter its emotional impact^3^ It is an adaptive coping strategy where one changes their perspective regarding a situation in order to reduce negative emotions or to enhance positive ones
Acceptance	An adaptive coping strategy characterized by an open and welcoming approach towards emotions, thoughts, or external events^4^
Rumination	Perseverative thinking or dwelling on negative thoughts or feelings without seeking an active solution^5^
Suppression	An emotion regulation strategy involving the conscious inhibition of emotionally expressive behaviour when emotionally aroused^6^
Distraction	The diversion of attention away from the emotion-eliciting event^7^
Perceived Stress	The appraisal of an event as stressful^8^
Allostatic Load	The cumulative physiological burden in response to chronic stress exposure and the body’s attempt to adapt to it over time^9^
Negative Affect	The experience of distress and negative emotional states such as sadness, anxiety, anger, guilt, shame, and disgust^10^
Avoidance	An individual’s attempts to deny and minimize thoughts, feelings, and bodily experiences in response to negative emotions^11^

Note: ^1^Lazarus & Folkman (1984)^39^; ^2^Ludwig et al. (2020a)^40^; ^3^Gross (1998)^41^; ^4^Hayes (1999)^42^; ^5^Nolen-Hoeksema et al. (2008)^43^; ^6^Gross & Levensen (1993)^44^; ^7^Grezellschak, Lincoln, and Westermann (2015)^45^; ^8^Cohen et al. (1983)^46^; ^9^McEwen & Stellar (1993)^47^; ^10^Watson & Clark (1988)^48^; ^11^Udachina et al. (2014)^49^.

Most studies examining coping across the psychosis continuum have employed cross-sectional designs, limiting insight into temporal relationships between coping strategies and clinical outcomes. Only one longitudinal study to date has examined coping in individuals with FEP.^13^ This study examined coping strategies and personality traits in 527 patients with recent-onset psychosis, with follow-up data collected between one and five years for 149 participants. These authors found that the coping strategy Reassuring Thoughts was associated with better baseline functioning, but neither coping nor personality traits predicted symptomatic remission or functioning at follow-up. These findings therefore highlight a gap in understanding the longitudinal role of coping strategies in FEP, particularly with respect to their associations with relapse, symptom trajectories, and functional outcomes. Machine Learning (ML) approaches have shown promise in predicting clinical outcomes in psychosis, including early symptom remission,^14^ one-year outcome trajectories,^15^ and long-term symptomatic and functional prognosis,^16^ as well as transition to psychosis in CHR samples.^17^ However, the extent to which coping strategies contribute to predictive models of relapse and clinical trajectories in FEP remains unclear. The current study addresses this gap by examining whether baseline coping styles are associated with subsequent relapse, positive symptom severity, and functional outcomes in individuals with FEP over a 12-month period, and by evaluating whether the inclusion of coping variables improves the performance of ML-based predictive models.

### Aims

The primary objective was to examine whether baseline coping style—including both maladaptive and adaptive strategies—predicts relapse, positive symptoms of psychosis, and functioning over 12 months. The specific objectives of this study were:

1. To examine whether maladaptive coping is associated with clinical and functional outcomes (relapse, positive symptoms, and functioning) within 12 months following baseline assessment.
2. To examine whether adaptive coping is associated with clinical and functional outcomes (relapse, positive symptoms, and functioning) within 12 months.
3. To evaluate the accuracy of machine learning models using coping style data to predict psychosis relapse at the individual level at 12 months follow-up.

It was hypothesized that maladaptive coping would be associated with greater relapse risk and poorer functioning (Hypothesis 1), while adaptive coping would be associated with lower relapse risk and better functioning (Hypothesis 2). Finally, it was hypothesized that machine learning models using coping style data would predict psychosis relapse within 12 months at the individual level with statistically significant accuracy (Hypothesis 3).

## Method

This study was a part of the Social Mind project at King’s College London, which examines the association between social stress and clinical outcomes in First Episode Psychosis. Ethical approval was granted by the London – Surrey Research Ethics Committee (20/LO/0331).

### Participants

A total of 269 FEP individuals, aged between 18 and 40 years, were recruited from 10 National Health Service (NHS) sites across the UK. All participants were outpatients recruited from Early Intervention Services. All participants met diagnostic criteria for FEP, as assessed using the Structured Clinical Interview for DSM-IV (SCID-I). Inclusion criteria required participants to be over 18 years of age and to be fluent in English. Exclusion criteria included having experienced the onset of psychosis more than 24 months before study entry, experienced more than one episode of psychosis, inability to provide informed consent, and/or an estimated IQ below 70 as determined from the treating clinicians/medical records.

### Study Design and Procedure

This longitudinal study involved structured clinical interviews conducted with participants at baseline, 4 months, 8 months, and 12 months online via MS Teams. Written informed consent was obtained at baseline. Participants received a voucher/bank payment as compensation for each interview completed. If an interview was missed, it was rescheduled where possible; alternatively, clinical records were consulted to gather data on symptoms and relapse events. The present study was purely observational and longitudinal; participants were not assigned to any intervention or treatment condition.

### Demographics

Age, gender, ethnicity, employment status, and education level was collected through clinical records and during face-to-face clinical interviews.

### Study Outcomes

#### Coping Style

The Brief COPE^18^ is a 28-item self-report questionnaire designed to assess coping responses to stressful experiences over the past two weeks. It consists of 14 subscales, each containing two items, rated on a 4-point Likert scale ranging from 1 (“I haven’t been doing this at all”) to 4 (“I’ve been doing this a lot”). Subscale scores were calculated by summing the two items corresponding to each domain (range 2-8). Composite scores were then created by summing the relevant subscales to represent *Adaptive Coping* (active coping, planning, reframing, acceptance, emotional support, instrumental support, humour, and religion) and *Maladaptive Coping* (self-distraction, denial, substance use, behavioural disengagement, venting, and self-blame). Higher scores indicate greater use of the respective coping style. The Brief COPE has demonstrated acceptable internal consistency and construct validity^25^ and has been successfully used in psychosis and CHR populations.^8^

#### Relapse

Relapse over the 12-month period was assessed using a single item from the study assessment pack (“Has the participant experienced a relapse since the last assessment?”)*.* Responses were recorded as a binary variable (yes or no). In line with Early Intervention in Psychosis (EIP) service criteria, relapse was defined as either re-hospitalisation or a clinically significant exacerbation of psychosis symptoms requiring increased clinical intervention (eg, medication adjustment or intensified community support). Information regarding relapse was obtained during participant interviews and corroborated using electronic clinical records to enhance accuracy. Participants were not required to be in full or partial remission at baseline; all met criteria for a first episode of psychosis and were receiving outpatient care at study entry.

#### Positive Symptoms

The Structured Clinical Interview for DSM-IV Axis I Disorders (SCID-I)^19^ is a semi-structured diagnostic interview designed to assess major psychiatric disorders according to DSM-IV criteria. As the SCID-I psychosis module assesses the presence of symptoms since the previous follow-up rather than providing symptom-specific severity ratings, outcomes were analysed as binary indicators of hallucinations and delusions (yes/no). A “yes” outcome for delusions was recorded if any of 12 items related to delusional themes (eg, persecutory, grandiose, thought broadcasting) were endorsed. Hallucinations were rated as present if any of four items were endorsed (eg, auditory, visual, tactile, other hallucination). Since positive symptoms are a key driver of relapse in psychosis, we also examined whether coping styles predicted hallucinations and delusions.

#### Functioning

The Global Assessment of Functioning (GAF) scale^20^ is a clinician-rated scale that provides a global measure of psychological, social, and occupational functioning. It includes two separate 0–100 scales: one assessing symptom severity (GAF Symptoms) and the other assessing functional impairment (GAF Disability), with higher scores indicating better functioning on both scales.

### Analyses

#### Regression-Based Statistical Analysis

The regression-based statistical analysis was conducted using STATA MP.18.0. Linear regressions were employed for baseline stress coping styles and GAF scores over time, while logistic regressions were used for binary outcomes such as relapse and SCID symptoms (hallucinations and delusions) based on baseline coping styles over 12 months. All regression models controlled for baseline demographic variables, including age, gender, ethnicity, education, and baseline employment status. Previous research has shown that coping strategies may vary across demographic and cultural groups, with cultural background and ethnicity influencing the use of adaptive and maladaptive coping in response to stress.^21^^,^^22^ These variables were therefore included to account for potential differences in coping patterns across participants. Baseline employment status was included as a covariate to control for its potential influence on coping and psychosocial outcomes^23^; however, in GAF models this may partially overlap with the occupational component of functioning, potentially leading to slight overadjustment.

To address potential bidirectionality between coping and symptoms, we conducted sensitivity analyses that adjusted for the baseline presence of the relevant positive symptom outcome (ie, baseline hallucinations in the hallucination models and baseline delusions in the delusion models). For relapse models, we additionally adjusted for baseline positive symptoms (ie, hallucinations and delusions). Missing data were marked and excluded for all regressions. The significance level was set to a standard of *P* < .05 for all statistical tests.

#### Machine Learning Analysis

Machine learning models were applied specifically to predict relapse, given its clinical significance and binary operationalisation, whereas continuous outcomes such as symptom severity and functioning would require alternative modeling approaches and larger samples to produce stable predictions. The data was randomly divided into training set and testing set at a ratio of 7:3. To address class imbalance in the binary outcome for relapse within 12 months (ratio of no relapse to relapse = 3.86:1), the Synthetic Minority Oversampling Technique (SMOTE)^24^ was applied exclusively to the training set to increase the representation of the minority class and reduce bias toward the majority class. CatBoost algorithm was employed for model development, with hyperparameter optimization performed using the Optuna framework.^25^ To ensure robustness, the optimization process was conducted with 5-fold cross-validation on the training set.

The final model’s performance was evaluated on the testing set using following metrics: accuracy (ACC), the receiver operating characteristic (ROC) curve, and the area under the ROC curve (AUC). The optimal threshold was determined by maximizing the Youden index. Variability in AUC and ACC were estimated via 1000 bootstrap resamples to obtain 95% confidence intervals (CIs) and the model was considered significantly better than chance if the 95% CI did not include the random-guessing level (0.5). Meanwhile, the DeLong test was employed to compare AUCs, while the one-tailed binomial test was applied to compare ACCs between models and against chance. All data preprocessing and modeling were conducted in Python (v3.10.16) using scikit-learn (v1.4.0), CatBoost (v1.2.8), and Optuna (v4.3.0) packages and statistical analysis was conducted in R (v4.4.3).

## Results

The demographic and clinical characteristics of the study sample are presented in Table 2. The regression analyses examining coping style as a predictor of relapse, positive symptoms, and functioning within 4-, 8, and 12-months follow-up are presented in Table 3.

**Table 2:** Demographic and Clinical Characteristics of FEP Participants.

**Characteristic**	**n (%) / Mean (SD)**		
**Demographic Variables**			
Age (years)	25.56 (5.28), n = 269		
Gender			
Male	151 (56%), n = 269		
Female	118 (44%), n = 269		
Ethnicity			
White	100 (37%), n = 269		
Black	75 (28%), n = 269		
Asian	45 (17%), n = 269		
Other	49 (18%), n = 269		
Employment Status			
Employed	135 (52%), n = 260		
Unemployed	125 (48%), n = 260		
Education Level			
Postgraduate	18 (7%), n = 266		
University	76 (29%), n = 266		
High School	131 (49%), n = 266		
Professional Training	28 (11%), n = 266		
Less than High School	13 (5%), n = 266		
**Clinical Characteristics**	**Full sample**	**Interview^†^**	**Electronic Health Records^†^**
**Relapse (n, %)**			
** Within** 4 Months	22 (9%), n = 256	17 (7%), n = 256	5 (2%), n = 256
** Within** 8 Months	35 (14%), n = 248	28 (11%), n = 248	7 (3%), n = 248
** Within** 12 Months	50 (20%), n = 245	34 (14%), n = 245	16 (6%), n = 245
**Hallucinations (n, %)**			
**Baseline**	200 (77%), n = 260	–	–
** Within** 4 Months	44 (20%), n = 221	41 (19%), n = 221	3 (1%), n = 221
** Within** 8 Months	57 (28%), n = 201	53 (26%), n = 201	4 (2%), n = 201
** Within** 12 Months	65 (34%), n = 194	50 (26%), n = 194	15 (8%), n = 194
**Delusions (n, %)**			
**Baseline**	247 (94%), n = 263	–	–
** Within** 4 Months	48 (22%), n = 220	42 (19%), n = 220	6 (3%), n = 220
** Within** 8 Months	69 (34%), n = 203	60 (30%), n = 203	9 (4%), n = 203
** Within** 12 Months	79 (41%), n = 195	60 (31%), n = 195	19 (10%), n = 195
**GAF – Disability (Mean, SD)**			
**Baseline**	71.78 (14.54), n = 262	–	–
4 Months	73.02 (16.41), n = 199	72.97 (16.56), n = 193	74.67 (11.29), n = 6
8 Months	74.26 (16.37), n = 180	74.49 (16.41), n = 173	68.43 (15.12), n = 7
12 Months	75.03 (16.53), n = 175	75.16 (16.86), n = 166	72.78 (8.41), n = 9
**GAF – Symptoms (Mean, SD)**			
**Baseline**	71.65 (14.68), n = 263	–	–
4 Months	74.01 (15.80), n = 200	73.96 (15.92), n = 194	75.67 (12.09), n = 6
8 Months	74.27 (16.48), n = 180	74.32 (16.59), n = 173	73.00 (14.32), n = 7
12 Months	75.73 (16.67), n = 175	75.84 (16.90), n = 166	73.56 (12.01), n = 9
**Maladaptive Coping: mean (SD), range**	22.77 (5.77), 12-47, n = 267	–	–
**Adaptive Coping: mean (SD), range**	42.75 (8.66), 23-62, n = 264	–	–

Note: *The total % and n may not reflect the full sample due to missing data, “Not sure,” or “Prefer not to say” responses.* ^†^Interview and EHR columns present values calculated using the same sample at each time point as the full sample column (ie, same n for percentages, means, and standard deviations).

**Table 3:** Coping Style as a Predictor of Relapse, Positive Symptoms, & Functioning within 4, 8, and 12 Months.

**Outcome**	**Time Point**	**Predictor**	**Estimate (OR/B)**	**β**	**SE**	**Z/t**	***P* value**	**95% CI (Lower-Upper)**
**Relapse**	4 months	Adaptive	0.98	–	0.03	−0.54	0.589	0.93-1.04
		Maladaptive	1.05	–	0.04	1.18	0.236	0.97-1.13
	8 months	Adaptive	1.00	–	0.02	−0.15	0.880	0.95-1.04
		Maladaptive	1.04	–	0.03	1.33	0.184	0.98-1.11
	12 months	Adaptive	1.01	–	0.02	0.31	0.757	0.97-1.05
		Maladaptive	1.06	–	0.03	2.12	0.034^*^	1.00-1.12
**Hallucinations**	4 months	Adaptive	0.96	–	0.02	−2.07	0.038^*^	0.92-1.00
		Maladaptive	1.08	–	0.03	2.60	0.009^**^	1.02-1.15
	8 months	Adaptive	0.96	–	0.02	−1.98	0.048^*^	0.93-1.00
		Maladaptive	1.07	–	0.03	2.21	0.027^*^	1.01-1.13
	12 months	Adaptive	0.97	–	0.02	−1.54	0.123	0.94-1.01
		Maladaptive	1.07	–	0.03	2.38	0.017^*^	1.01-1.13
**Delusions**	4 months	Adaptive	0.95	–	0.02	−2.38	0.018^*^	0.91-0.99
		Maladaptive	1.06	–	0.03	1.93	0.052^†^	1.00-1.12
	8 months	Adaptive	0.97	–	0.02	−1.76	0.079^†^	0.93-1.00
		Maladaptive	1.09	–	0.03	2.94	0.003^**^	1.03-1.15
	12 months	Adaptive	0.98	–	0.02	−1.27	0.205	0.95-1.01
		Maladaptive	1.07	–	0.03	2.49	0.013^*^	1.02-1.13
**GAF Disability**	4 months	Adaptive	0.35	0.19	0.13	2.73	0.007^**^	0.10-0.60
		Maladaptive	−0.36	−0.13	0.19	−1.86	0.064^†^	−0.74-0.02
	8 months	Adaptive	0.09	0.05	0.14	0.65	0.514	−0.18-0.36
		Maladaptive	−0.49	−0.17	0.21	−2.34	0.020^*^	−0.90-−0.08
	12 months	Adaptive	0.23	0.12	0.14	1.64	0.102	−0.05-0.52
		Maladaptive	−0.32	−0.11	0.22	−1.41	0.160	−0.76-0.13
**GAF Symptoms**	4 months	Adaptive	0.29	0.16	0.13	2.21	0.028^*^	0.03-0.55
		Maladaptive	−0.73	−0.27	0.19	−3.87	*P* < .001^***^	−1.11-−0.36
	8 months	Adaptive	0.12	0.06	0.14	0.81	0.418	−0.17-0.40
		Maladaptive	−0.60	−0.21	0.22	−2.79	0.006^**^	−1.03-−0.18
	12 months	Adaptive	0.09	0.05	0.15	0.65	0.517	−0.19-0.38
		Maladaptive	−0.50	−0.17	0.23	−2.22	0.028^*^	−0.95-−0.06

Key: ^*^P < .05, ^**^P < .01, ^***^P < .001*,*  ^†^Trend toward significance (*P* = .05–0.10). All results are adjusted for age, gender, ethnicity, employment status, and education level.

### Maladaptive Coping and Relapse

**Figure 1:**
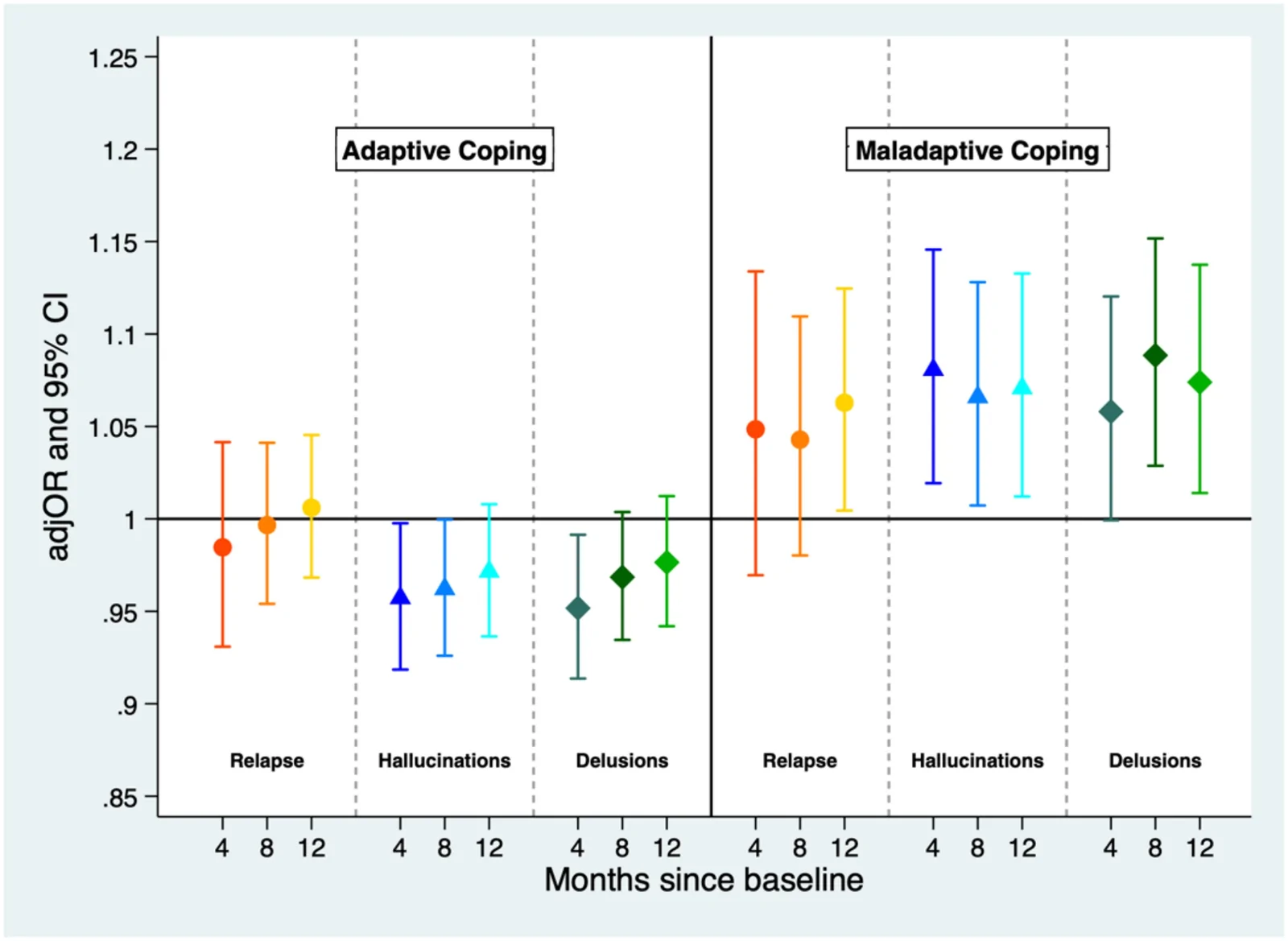
Association between coping style and relapse, hallucinations, & delusions within 4, 8, and 12 months. Note: adjusted odds ratios (adjOR) and 95% confidence intervals (CI) represent the change in odds of the outcome associated with a one-unit increase in maladaptive or adaptive coping strategy scores. An OR greater than 1 indicates higher odds of outcome per unit increase, while an OR less than 1 indicates lower odds. All models were adjusted for age, gender, ethnicity, employment status, and education level. Colours and shapes denote outcomes and follow-up timepoints: circles (red – yellow scale) represent relapse at 4, 8, and 12 months; triangles (dark – light blue scale) represent hallucinations at 4, 8 and 12 months; and diamonds (dark – light green scale) represent delusions at 4, 8, and 12 months

Logistic regression models examined the association between maladaptive coping styles and relapse within 4, 8, and 12 months (see Figure 1). Maladaptive coping was significantly associated with increased odds of relapse within 12 months (OR = 1.06, 95% CI: 1.00–1.12, *P* = .034) but not within 4 months (OR = 1.05, 95% CI: 0.97–1.13, *P* = .236), or 8 months (OR = 1.04, 95% CI: 0.98–1.11, *P* = .184).

As relapse in psychosis is commonly defined by the re-emergence or worsening of positive symptoms, we examined the association between coping and two core positive symptom domains: hallucinations and delusions. Maladaptive coping was significantly associated with increased odds of experiencing hallucinations within 4 months (OR = 1.08; 95% CI: 1.02–1.15; *P* = .009), 8 months (OR = 1.07; 95% CI: 1.01–1.13; *P* = .027), and 12 months (OR = 1.07; 95% CI: 1.01–1.13; *P* = .017). Maladaptive coping showed a non-significant trend-level positive association with the experience of delusions within 4 months (OR = 1.06, 95% CI: 1.00–1.12, *P* = .053), and was significantly associated with increased odds of experiencing delusions within 8 months (OR = 1.09, 95% CI: 1.03–1.15, *P* = .003) and 12 months (OR = 1.07, 95% CI: 1.02–1.13, *P* = .013).

Sensitivity analyses adjusting for baseline symptom status yielded largely similar results. However, the associations between maladaptive coping and hallucinations at 8 and 12 months were attenuated and no longer statistically significant, although the odds ratios and CIs were similar and the direction of the associations remained unchanged. All other results remained unchanged. Full results of these sensitivity analyses are presented in Supplementary Table 1.

### Maladaptive Coping and Functioning

Maladaptive coping showed a trend-level negative association with poorer (ie, lower) disability functioning within 4 months (B = −0.36, SE = 0.19, β = -0.13, *P* = .064, 95% CI [−0.74, 0.02]), and was significantly associated with poorer disability functioning within 8 months (B = −0.49, SE = 0.21, β = -0.17, *P* = .020, 95% CI [−0.90, −0.08]), but not within 12 months (B = −0.32, SE = 0.22, β = -0.11, *P* = .160, 95% CI [−0.76, 0.13]).

Maladaptive coping was significantly associated with poorer symptom functioning within 4 months (B = −0.73, SE = 0.19, β = -0.27, *P* < .001, 95% CI [−1.11, −0.36]), 8 months (B = −0.60, SE = 0.22, β = -0.21, *P* = .006, 95% CI [−1.03, −0.18]), and 12 months (B = −0.50, SE = 0.23, β = -0.17, *P* = .028, 95% CI [−0.95, −0.06]).

### Adaptive Coping and Relapse

Logistic regression models examined the association between adaptive coping styles and relapse within 4, 8, and 12 months (see Figure 1). Adaptive coping was not significantly associated with lower odds of relapse within 4 months (OR = 0.98, 95% CI: 0.93–1.04, *P* = .589), 8 months (OR = 1.00, 95% CI: 0.95–1.04, *P* = .880), or 12 months (OR = 1.01, 95% CI: 0.97–1.05, *P* = .757). Adaptive coping was significantly associated with lower odds of experiencing hallucinations within 4 months (OR = 0.96; 95% CI: 0.92–1.00; *P* = .038), and 8 months (OR = 0.96; 95% CI: 0.93–1.00; *P* = .048) but by 12 months this effect was non-significant (OR = 0.97; 95% CI: 0.94–1.01; *P* = .123). Adaptive coping was significantly associated with lower odds of experiencing delusions within 4 months (OR = 0.95; 95% CI: 0.91–0.99; *P* = .018), showed a trend-level association with lower odds within 8 months (OR = 0.97; 95% CI: 0.93–1.00; *P* = .079), but by 12 months this effect was non-significant (OR = 0.98; 95% CI: 0.94–1.01; *P* = .205).

### Adaptive Coping and Functioning

Adaptive coping was significantly associated with better disability scores within 4 months (B = 0.35, SE = 0.13, β = 0.19, *P* = .007, 95% CI [0.10, 0.60]), but not within 8 months (B = 0.09, SE = 0.14, β = 0.05, *P* = .514, 95% CI [−0.18, 0.36]) or 12 months (B = 0.23, SE = 0.14, β = 0.12, *P* = .102, 95% CI [−0.05, 0.52]).

Adaptive coping was significantly associated with better symptom functioning within 4 months (B = 0.29, SE = 0.13, β = 0.16, *P* = .028; 95% CI [0.03, 0.55]); but not within 8 months (B = 0.12, SE = 0.14, β = 0.06, *P* = .418, 95% CI [−0.17, 0.40]) or 12 months (B = 0.09, SE = 0.15, β = 0.05, *P* = .517, 95% CI [−0.19, 0.38]).

### Individual-Level Predictions

**Figure 2:**
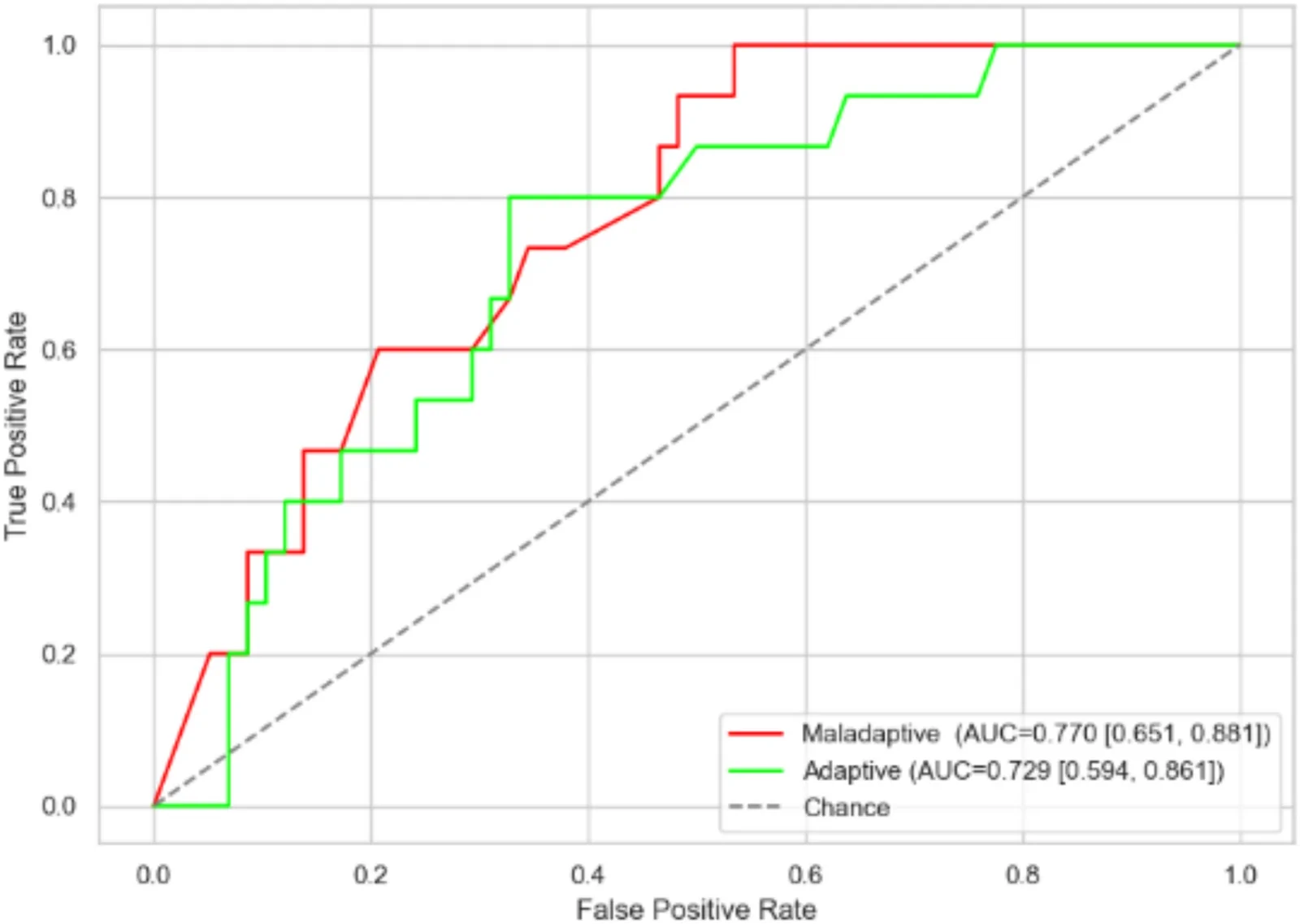
ROC Curves for Machine Learning Models Using Maladaptive & Adaptive Coping (AUC Values and 95% Confidence Intervals Are Reported in the Legend)

**Figure 3:**
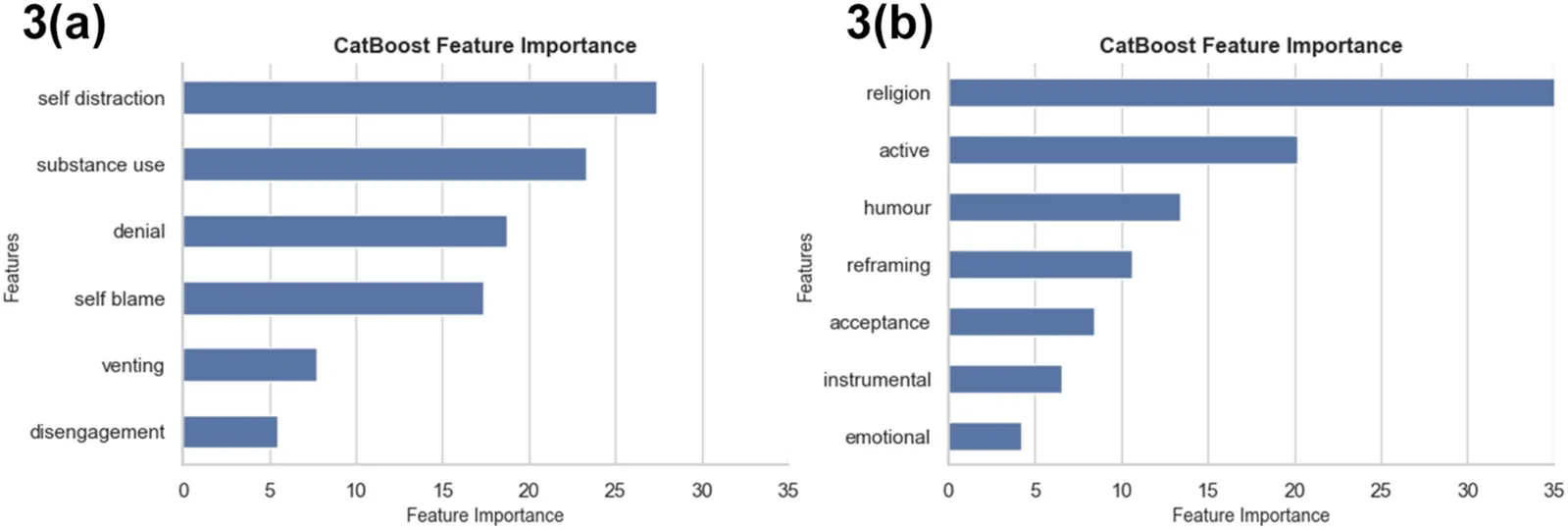
(a) Maladaptive Coping Strategies Driving Prediction of Relapse within 12 Months; (b) Adaptive Coping Strategies Driving Prediction of Relapse within 12 Months

Next, we examined whether maladaptive and adapting coping significantly predicted relapse at the individual level and further examined which specific components contributed to these effects. Machine learning models demonstrated that both maladaptive and adaptive coping strategies significantly improved the prediction of relapse within 12 months, with maladaptive coping showing stronger predictive performance (AUC = 0.77 [0.651,0.881], *P* < .001; accuracy = 0.75 [0.658, 0.849], *P* < .001) compared to adaptive coping (AUC = 0.73 [0.594, 0.861], *P* = .017; accuracy = 0.70 [0.589, 0.795], *P* < .001) (Figure 2). Within maladaptive coping, self-distraction and substance use were the two most important predictors of relapse (Figure 3a). Among the adaptive coping strategies, spiritual/religious coping and active coping contributed most strongly to prediction accuracy (Figure 3b).

## Discussion

This longitudinal study examined whether baseline coping styles were associated with clinical and functional outcomes in FEP over 12 months. Maladaptive coping was significantly associated with higher relapse rates within 12 months (OR = 1.06), corresponding to ~6% increase in relapse odds for each point increase in maladaptive coping. This association was not observed within 4 or 8 months, likely due to increased statistical power and cumulative psychosocial stressors over the longer timeframe. Although the overall sample size decreased due to attrition, the number of relapse events accumulated across the follow-up period, thereby increasing the statistical power to detect predictors of relapse within 12 months. Adaptive coping was not associated with reduced relapse risk at group level, suggesting that maladaptive strategies may be more strongly associated with relapse. These findings align with contemporary cognitive models of psychosis, namely the *Bio-Psychosocial Model of Transition to Psychosis*,^26^ which emphasize the role of stress, core belief re-activation, negative affect, cognitive biases, and maladaptive coping as factors implicated in psychosis symptom formation and relapse.

The present findings demonstrate that maladaptive coping at baseline was associated with a higher likelihood of relapse and the persistence of positive symptoms over 12 months in FEP. Although longitudinal evidence is limited, cross-sectional studies in schizophrenia indicate that negative coping correlates with relapse, while positive coping is associated with lower relapse risk.^27^ Together, these findings suggest that maladaptive coping may be associated with increased relapse risk and symptom persistence in FEP. These findings highlight the need for further prospective examination of baseline coping in FEP.

Maladaptive coping was significantly associated with hallucinations at all timepoints (OR = 1.07 – 1.08), corresponding to ~7%-8% increase in odds of experiencing hallucinations for each point increase in maladaptive coping. Maladaptive coping was also significantly associated with delusions within 8 and 12 months (OR = 1.07 – 1.09), corresponding to ~7%-9% increase in odds of delusions per point increase. These findings support the consistent association between maladaptive coping and positive symptoms in FEP. This aligns with longitudinal CHR evidence showing that maladaptive coping at baseline was associated with more severe positive and negative symptoms over time.^8^ In the present study, adaptive coping was associated with reduced odds of hallucinations (4 and 8 months) and delusions (4 months), but these effects were not sustained, suggesting an early, transient association with lower symptom levels. These findings highlight that while adaptive coping may initially be associated with lower symptom severity, sustaining these strategies may require ongoing support, consistent with evidence that FEP individuals use adaptive coping less consistently than controls.^28^ Although the observed odds ratios were modest on a per-point basis, the range of coping scores in the sample suggests that larger differences in coping style may correspond to clinically meaningful differences in outcomes. For example, a one standard deviation increase in maladaptive coping in this sample (≈ 6 points) would correspond to ~1.4-fold higher odds of relapse or positive symptoms.

Maladaptive coping was associated with lower GAF disability scores within 8 months (B = -0.49), with each one-point increase corresponding to ~0.5-point decrease in GAF scores. Maladaptive coping was also associated with poorer GAF symptom scores at all time points, indicating a persistent association with poorer functioning. Adaptive coping was associated with better scores across both GAF domains within 4 months only, suggesting the possibility that sustained support may be required to maintain these associations over time. Longitudinal CHR research shows that adaptive coping was associated with better social functioning over time,^8^ while SZ studies indicate that maladaptive coping was associated with lower quality of life and impaired functioning.^29^^,^^30^ Together, these findings suggest that coping strategies may be associated not only with symptom trajectories but also with functional outcomes across the psychosis continuum.

Machine learning models identified both maladaptive and adaptive coping styles as significant features associated with relapse prediction. While maladaptive coping demonstrated higher predictive accuracy, adaptive coping also played a meaningful role. Among maladaptive strategies, self-distraction and substance use emerged as key predictive features of relapse risk. In contrast, active coping and spiritual/religious coping were influential within the adaptive domain. These findings suggest that maladaptive coping may be more strongly and immediately associated with relapse risk, whereas adaptive coping strategies may be associated with lower relapse likelihood depending on sustained support or specific contextual factors. This aligns with the broader literature showing that specific coping strategies, such as emotion-focused or avoidant coping, are particularly linked to positive symptoms and negative affect in psychosis.^28^^,^^31^^,^^32^ While substance use was classified as a maladaptive coping strategy in our analyses, it differs from other coping behaviours in that it involves direct neurobiological effects that may be associated with more severe or persistent psychosis symptoms. Furthermore, substance use may occur both as a coping response to stress and as part of a comorbid substance use disorder. Given that substance use accounted for a substantial portion of the observed effects of maladaptive coping, these findings should be interpreted with caution. Future research would benefit from distinguishing between coping-related substance use and persistent substance use associated with substance use disorders in order to clarify its distinct role in psychosis outcomes. Clinically, the present findings highlight the potential value of addressing maladaptive behaviours while concurrently promoting adaptive strategies, particularly problem-solving and faith-based approaches that resonate with clients’ personal values. This aligns with existing evidence that spirituality and religiosity can contribute meaningfully to recovery in psychosis.^33^

In summary, these findings underscore the strong association between maladaptive coping and psychosis symptom severity and functional impairment in FEP. The continuity of maladaptive coping patterns across CHR, FEP, and SZ highlights the potential value of early coping-focused interventions, which may be associated with improvements in symptom trajectories, functional outcomes, and relapse rates. Maladaptive coping may be associated with higher relapse risk through patterns characterized by avoidance behaviours, reduced engagement with adaptive problem-solving, heightened stress sensitivity, negative affect, and cognitive biases.^26^^,^^32^^,^^34^^,^^35^ Interventions that target both the reduction of maladaptive coping and the promotion of adaptive coping, such as problem-focused and spiritual/religious strategies, may be associated with improved recovery outcomes. Machine learning approaches offer further value in identifying subtle risk patterns that may support the development of more tailored early intervention strategies.

### Strengths and Limitations

This is the first study to examine the temporal association between baseline coping, with relapse, positive symptoms of psychosis, and functioning in an FEP sample in a 12-month longitudinal study. It employed a mixture of linear and logistic regressions, as well as advanced techniques of machine learning to examine the associations between coping styles and clinical and functional outcomes in FEP, which is a strength of the present study.

In terms of limitations, there was some sample attrition from baseline (n = 269) to the 12-month follow-up (n = 245), which may have reduced statistical power for analyses of symptom severity and functional outcomes. However, for relapse, the accumulation of events across the 12-month period increased statistical power to detect associations with baseline coping strategies despite this attrition. Importantly, coping strategies were assessed only at baseline, which precluded examination of potential changes in coping over time, such as those resulting from psychological interventions, and their influence on symptomatic or functional outcomes. Future studies incorporating repeated assessments of coping would help clarify how coping strategies evolve over time and across illness stages. Notably, some evidence suggests that coping styles in individuals with, or at clinical high risk for, psychotic disorders may vary depending on illness stage.^24^

While baseline employment status was included as a covariate to account for potential confounding, we acknowledge that controlling for employment in GAF models may partially overlap with the occupational component of functioning, potentially leading to some overadjustment.

Although relapse was defined using standard EIP service criteria and corroborated through clinical records, it was operationalized as a binary variable and may not fully capture variation in relapse severity or type (eg, hospitalisation versus community-managed exacerbation of psychosis symptoms).

Moreover, our study used binary outcomes for hallucinations and delusions over the follow-up period, which does not capture more subtle variations in symptom severity or residual psychosis experiences. Consequently, the influence of mild or subthreshold symptoms on functioning and coping strategies may not be fully reflected in our analyses.

### Clinical Implications

The finding that maladaptive coping at baseline is significantly associated with relapse within 12 months highlights the importance of assessing and addressing coping styles in Cognitive Behavioural Therapy for Psychosis (CBTp) within Early Intervention Services. Targeting both the reduction of maladaptive coping and the promotion of adaptive coping may represent clinically meaningful intervention strategies associated with symptomatic and functional recovery and reduced relapse risk in FEP. Specifically, our findings suggest the potential value of targeting self-distraction and substance misuse, while promoting active problem-solving and incorporating spiritual or religious coping where appropriate. While baseline adaptive coping predicted outcomes at 4–8 months, this association was weaker at 12 months. Given that coping was assessed only at baseline, this likely reflects the decreasing predictive utility of a single baseline measure over longer follow-up periods rather than a decline in the benefit of coping itself. Coping is a dynamic construct that may change over time, and future interventions may benefit from repeated assessment and ongoing support of adaptive coping strategies throughout the early course of illness. Collectively, these findings suggest that early interventions that explicitly reduce maladaptive coping and strengthen adaptive coping may be associated with improved symptomatic and functional outcomes in FEP.

### Future Directions

Future studies employing machine learning approaches could examine which components of maladaptive coping are associated with specific positive symptom subtypes (ie, command hallucinations vs persecutory delusions),^35^^,^^36^ as well as which aspects of adaptive coping are associated with better outcomes. Future research could also explore associations between coping and cognitive processes such as cognitive biases and core beliefs to further clarify their relationship across the psychosis spectrum (ie, CHR, FEP, and SZ).^35^^,^^37^ Given that baseline coping may become a weaker predictor of outcomes over longer follow-up periods, longitudinal studies with repeated coping assessments could explore whether interventions that reduce maladaptive coping and reinforce adaptive coping over time (eg, at monthly intervals) lead to sustained improvements in clinical and functional outcomes in FEP.

Moreover, an important distinction has emerged regarding the concept of distraction, whereby distraction combined with acceptance has been associated with improved well-being, while distraction combined with avoidance has been linked to poorer outcomes.^38^ This highlights the need for future research to differentiate between these forms of distraction and examine their distinct effects on outcomes.

Future studies would also benefit from employing both quantitative and qualitative methods to examine coping responses separately in relation to stress, negative affect, and psychosis symptoms. Coping strategies may differ depending on whether an individual is addressing external stressors, emotional distress, or internal psychosis experiences (eg, auditory hallucinations versus persecutory delusions). Elucidating these domain-specific coping mechanisms would facilitate the development of more precise, context-sensitive interventions in FEP.

Ecological Momentary Assessment (EMA) methods could further elucidate coping responses and triggers in real time, providing a mechanistic understanding of how coping unfolds in daily life among individuals with FEP. Future studies may also benefit from examining coping strategies not captured by the Brief COPE, such as suppression, rumination, avoidance, and both avoidant and acceptance-based forms of distraction, to better understand their roles in relapse and psychosis symptomatology. Finally, incorporating continuous measures of positive symptom severity across extended follow-up intervals may allow a more nuanced understanding of how coping strategies relate to the full spectrum of psychotic experiences, including mild or subthreshold symptoms that may persist during periods of clinical remission.

### Conclusion

This study highlights the importance of incorporating coping assessments into routine clinical care and the value of tailoring interventions to target specific maladaptive coping behaviours, such as self-distraction and substance use, which were significantly associated with relapse in FEP. Conversely, promoting adaptive strategies such as active problem-solving and faith-based coping when aligned with a client’s values may be associated with better recovery outcomes in FEP. By demonstrating associations between coping styles and relapse, positive symptoms, and functioning, this study underscores the relevance of coping as a modifiable treatment target in FEP and provides direction for future intervention development and research.

## Supplementary Material

Coping_paper_Supplementary_Material_FINAL_200326_sbag079

## Data Availability

The data generated and analysed for the current study are not publicly available in accordance with ethical and institutional guidelines, but de-identified data may be made available from the corresponding author on reasonable request.

## Notes

**Dr Anna Georgiades** (PhD, DClinPsy, BABCP, FHEA) is a Senior Lecturer in Early Intervention in Psychosis Education and Deputy Programme Director for the Early Intervention in Psychosis MSc at the Institute of Psychiatry, Psychology, and Neuroscience (IoPPN) at King’s College London. She is also a Principal Clinical Psychologist for the Brent Early Intervention Service in the National Health Service (NHS).
