# Supplementary material for: Coping Style as a Predictor of Relapse and Functioning in First Episode Psychosis
Source: Schizophr Bull. 2026 Aug 24;52(5):sbag079. doi: 10.1093/schbul/sbag079 (PMC13502908; doi:10.1093/schbul/sbag079)
Supplement: Supplementary file 1 [file coping_paper_supplementary_material_final_200326_sbag079.docx]

**SUPPLEMENTARY MATERIAL**

**Supplementary Table 1. Sensitivity Analysis of Coping Style as a Predictor of Relapse and Positive Symptoms adjusted for Baseline Positive Symptoms.**

| **Outcome** | **Time Point** | **Predictor** | **Estimate (OR/B)** | **SE** | **Z/t** | **p value** | **95% CI (Lower-Upper)** |
| --- | --- | --- | --- | --- | --- | --- | --- |
| **Relapse** | 4 months | Adaptive | 0.98 | 0.03 | -0.54 | 0.591 | 0.93-1.04 |
|  |  | Maladaptive | 1.05 | 0.04 | 1.32 | 0.188 | 0.97-1.15 |
|  | 8 months | Adaptive | 1.00 | 0.02 | -0.17 | 0.868 | 0.95-1.04 |
|  |  | Maladaptive | 1.05 | 0.03 | 1.45 | 0.148 | 0.98-1.12 |
|  | 12 months | Adaptive | 1.01 | 0.01 | 0.29 | 0.775 | 0.96-1.04 |
|  |  | Maladaptive | 1.07 | 0.03 | 2.15 | 0.031* | 1.00-1.13 |
| **Hallucinations** | 4 months | Adaptive | 0.95 | 0.02 | -2.16 | 0.031* | 0.91-1.00 |
|  |  | Maladaptive | 1.06 | 0.03 | 1.99 | 0.046* | 1.00-1.13 |
|  | 8 months | Adaptive | 0.96 | 0.02 | -1.90 | 0.057† | 0.93-1.00 |
|  |  | Maladaptive | 1.05 | 0.03 | 1.67 | 0.095† | 0.99-1.11 |
|  | 12 months | Adaptive | 0.97 | 0.02 | -1.29 | 0.198 | 0.94-1.01 |
|  |  | Maladaptive | 1.06 | 0.03 | 1.94 | 0.053† | 1.00-1.12 |
| **Delusions** | 4 months | Adaptive | 0.95 | 0.02 | -2.41 | 0.016* | 0.91-0.99 |
|  |  | Maladaptive | 1.06 | 0.03 | 1.82 | 0.069† | 1.00-1.12 |
|  | 8 months | Adaptive | 0.97 | 0.02 | -1.85 | 0.065† | 0.93-1.00 |
|  |  | Maladaptive | 1.09 | 0.03 | 2.83 | 0.005** | 1.03-1.15 |
|  | 12 months | Adaptive | 0.98 | 0.02 | -1.36 | 0.173 | 0.94-1.01 |
|  |  | Maladaptive | 1.07 | 0.03 | 2.38 | 0.017* | 1.01-1.13 |

*Key: *p* < 0.05*, **p* < 0.01*, ***p* < 0.001*,* †Trend toward significance (p = 0.05–0.10).

*Relapse results are adjusted for age, gender, ethnicity, employment status, education level, presence of delusions and hallucinations at baseline.*

*Hallucinations results are adjusted for age, gender, ethnicity, employment status, education level, presence of hallucinations at baseline.*

*Delusions results are adjusted for age, gender, ethnicity, employment status, education level, presence of delusions at baseline.*
